# The International Federation for Emergency Medicine report on emergency department crowding and access block: A brief summary

**DOI:** 10.1136/emermed-2020-210716

**Published:** 2021-01-13

**Authors:** Arshia P Javidan, Kim Hansen, Ian Higginson, Peter Jones, Eddy Lang, International Federation Emergency Department Crowding and Access Block Task Force, Arshia Javidan

**Affiliations:** 1 Faculty of Medicine, University of Toronto, Toronto, Ontario, Canada; 2 Institute of Health Policy, Management, and Evaluation, University of Toronto, Toronto, Ontario, Canada; 3 Emergency Department, Prince Charles Hospital, Chermside, Queensland, Australia; 4 Emergency Department, St. Andrew's War Memorial Hospital, Brisbane, Queensland, Australia; 5 Emergency Department, Derriford Hospital, Plymouth, Plymouth, UK; 6 Emergency Department, Auckland City Hospital, Auckland District Health Board, Auckland, New Zealand; 7 Department of Surgery, University of Auckland, Auckland, New Zealand; 8 Department of Emergency Medicine, Cumming School of Medicine, University of Calgary, Calgary, Alberta, Canada; 9 Department of Emergency Medicine, Alberta Health Services, Calgary, Alberta, Canada

**Keywords:** care systems, crowding, emergency care systems, emergency department management, emergency department operations

## Abstract

**Objective:**

To develop comprehensive guidance that captures international impacts, causes and solutions related to emergency department (ED) crowding and access block.

**Methods:**

Emergency physicians representing 15 countries from all International Federation of Emergency Medicine (IFEM) regions composed the Task Force. Monthly meetings were held via video-conferencing software to achieve consensus for report content. The report was submitted and approved by the IFEM Board on June 1, 2020.

**Results:**

A total of 14 topic dossiers, each relating to an aspect of ED crowding, were researched and completed collaboratively by members of the Task Force.

**Conclusions:**

The IFEM report is a comprehensive document intended to be used in whole or by section to inform and address aspects of ED crowding and access block. Overall, ED crowding is a multifactorial issue requiring systems-wide solutions applied at local, regional, and national levels. Access block is the predominant contributor of ED crowding in most parts of the world.

Emergency department (ED) crowding and access block represent potentially the greatest threats to the core mission of emergency care across the world. The problem is pervasive, massive in scale, and amounts to a public health emergency with potentially lethal consequences.^1^ At its core, crowding and access block overwhelm ED resources and prevent the delivery of timely and effective care for patients. These are patients in need of necessary and immediate attention for the whole range of medical, trauma and behavioural emergencies that can impact a person or community. The causes of ED crowding and access block are complex and multifactorial and can vary considerably not only between hospitals, jurisdictions, and countries, but also within the same setting during different periods of time.^1 2^

The International Federation of Emergency Medicine (IFEM) recognised that there was both an extreme need and a unique opportunity to provide EDs around the world with expert and evidence-based guidance. Recognising crowding and access block were wicked problems (problems that are challenging to solve due to complexity, breadth, and/or contradictory elements) requiring adaptive solutions, the plan was to develop a resource that could be adapted to local circumstances. The ED Crowding and Access Block Task Force was constructed with this goal and endorsed by the IFEM Board and launched at the International Conference on Emergency Medicine conference in South Korea in 2019. Since that time, the ED Crowding and Access Block Task Force Terms of Reference were approved, and the task force has seen involvement from all IFEM regions. Over 30 emergency medicine (EM) physician experts and thought leaders, with a broad range of expertise, have been joining monthly video conferences and contributing to fourteen distinct dossiers and well-referenced synopses which constitute the basis for this report (box 1).

Box 1 **Overview of breadth of dossier topics found in the International Federation of Emergency Medicine Emergency Department Crowding and Access Block Task Force Report**

**Dossier topic**

**Background**

- Evidence base for effects of crowding.
- Financial and human costs of crowding.
- Metrics.
- International experience.
- Case studies and patient voices.

**Patient flow**

- Emergency medical services (prehospital services) offload.
- Input and demand management.
- Throughput.
- Output and boarding.

**Management**

- Leadership.
- Legal risks and regulatory violations.
- Policy.
- Advocacy.
- Early lessons from COVID-19 and disaster medicine.

This report examines the complexity of ED Crowding and Access Block through multiple important lenses. These range from an accounting of the impacts of the problem through to tactical and strategic solutions including policy, advocacy, operational and ‘on-the-floor’ initiatives. One overwhelmingly common theme that emerged through Task Force deliberations is that the problem may be misnamed. ED Crowding and Access Block is not an issue isolated to the ED, but fundamentally a health systems issue.^1 3^ Emergency Departments are well prepared to serve as the ‘safety net’ for a wide range of medical, traumatic and behavioural emergencies, however, EDs cannot fulfil this mission if they are also forced to become the ‘safety valve’ for dysfunction and limited capacity within the community and the hospital. Despite this, the Task Force would also share the view that EDs that are not contributing to solutions for healthcare system dysfunction are also part of the problem; hence the vital importance of emergency care providers who are well versed in system issues to infiltrate decision making and public awareness realms at multiple levels.

As a ‘wicked problem’ for healthcare systems internationally, experts and thought leaders around the world have invested a remarkable amount of resources to understand the problem and formulate solutions. This report is designed to leverage that vast international experience and serve as a comprehensive global resource for EDs facing the challenge of crowding and access block. This document is meant to be used as a toolbox with each section acting as one tool of many to diagnose and treat an unsafe and overwhelmed ED. Leaders in emergency care will be able to use these instruments to address their local circumstances both on a short-term and long-term basis. This report is also meant to be shared in portion, or in its entirety, with all of the stakeholders that can be impacted by ED crowding and access block as well as the partners necessary to mitigate and distribute risk and allow emergency care to fulfil its core mission.

The report, including the full list of authors, can be found in its entirety on the IFEM website.^4^
