# Correction: *The International Federation for Emergency Medicine report on emergency department crowding and access block: A brief summary*

**DOI:** 10.1136/emermed-2020-210716corr1

**Published:** 2021-09-29

Javidan AP, Hansen K, Higginson I *et al*. The International Federation for Emergency Medicine report on emergency department crowding and access block: A brief summary. *Emerg Med J* 2021;38:245–46.

Since first publication the provenance and peer review statement has been added to this article.

